# Special Issue “Molecular Studies of Dermatitis: From Mechanism to Therapy”

**DOI:** 10.3390/ijms25052696

**Published:** 2024-02-26

**Authors:** Tokio Nakada

**Affiliations:** Department of Dermatology, Showa University Fujigaoka Hospital 1-30, Fujigaoka, Aoba-ku, Yokohama 227-8501, Kanagawa, Japan; tokio@med.showa-u.ac.jp

Dermatitis (eczema) represents a group of inflammatory cutaneous diseases. Acute eczema demonstrates various rashes: erythema, papules, vesicles, pustules, crusts, erosion, clinical scaling, and histopathological spongiosis. On the other hand, chronic eczema reveals lichenification and histopathological acanthosis. This group includes the following diseases: contact, atopic, seborrheic, and stasis dermatitis; hand, nummular, and asteatotic eczema, etc. Although most of these are common diseases in clinical practice, their pathogenesis has not been fully clarified yet. However, recent studies have gradually provided new insights.

Contact dermatitis is classified into three types: irritant contact, allergic contact, and photocontact dermatitis. It has long been believed that irritant contact dermatitis is caused by a stimulus, while allergic contact dermatitis is a delayed-type hypersensitivity reaction involving T and antigen-presenting dendric cells. But recent studies have demonstrated that the innate immune system plays an important role in contact dermatitis. All types of contact dermatitis are initiated by the penetration of a chemical. Its epicutaneous stimulation causes keratinocytes to secrete cytokines, and inflammation may occur, depending on the innate immune system. Such damage-associated molecular patterns (DAMPs) play an important role in irritant contact dermatitis [1]. The innate immune system is also important for the sensitization and elicitation phases in allergic contact dermatitis [2]. DAMPs are recognized by pattern recognition receptors, including Toll-like and nucleotide-binding oligomerization domain-like receptors, in innate immune cells, and these cells cascade the signal to produce several cytokines and chemokines, including tumor necrosis factor (TNF)-α, interferon (IFN)-α, and interleukin (IL)-1β, IL-4, IL-6, IL-12, IL-13, IL-17, IL-18, and IL-23 [2]. In addition, it is interesting to examine the roles of mast cells, eosinophils, basophils, and macrophages in contact dermatitis. Gaudenzio et al. suggested that mast cells can amplify dermatitis of mild severity and can limit the inflammation and tissue injury associated with severe conditions [3]. Kim et al. reported that eosinophil cationic proteins released by eosinophils can be a potential surrogate marker of allergic contact dermatitis [4]. It was suggested that basophils act as antigen-presenting cells to drive Th2 cell differentiation and the basophil-specific effector molecules involved allergic responses [5]. Patch testing aims to reproduce an eczematous reaction by applying allergens via occlusion on the intact skin of patients suspected to have allergic contact dermatitis. Despite the limitations, this is by no means the cornerstone of the diagnostic procedure for such patients. The old dermatology textbooks describe that reading is performed 2 and 3 days after application. However, reading should be performed not only these days but occurring at day 7 [6]. There are also allergens that require reading after a longer period of time; many cases of late-onset positive reactions to gold sodium thiosulfate (GST) have been reported [7]. We studied such cases histologically and immunohistologically to elucidate the pathogenesis of these reactions. We speculate that late reactions to GST may be due to gold ion’s specificity as it penetrates into the skin and/or is by the immune system. In addition, very persistent positive reactions to gold have also been noted. This might be due to the presence of diffused gold ions, which can only slowly be removed [8]. Immunohistopathologically, the infiltrated cells were CD3+ cells, and CD20+ and CD79a+ B cells were interposed [8]. Photocontact dermatitis is produced by the combination of skin contact with a compound together with ultraviolet light. Most cases are now thought to be caused not by prohaptens, but by photohaptens, with long-wave ultraviolet A light (UVA) as their wavelength of action [9].

Atopic dermatitis is a disease characterized by relapsing eczema with pruritus. The International Study of Asthma and Allergies in Childhood (ISAAC) conducted a worldwide survey of atopic dermatitis from 1994 to 1996. The overall prevalence among 6-to-7-year-old children was 7.3%, ranging from 1.1% in Iran to 18.4% in Sweden, and this was 7.4% among those aged 13–14 years, ranging from 0.8% in Albania to 17.7% in Nigeria [10]. In this way, atopic dermatitis was thought to be a disease that mainly affected children, but it is now known to occur in people of all ages. Although its pathogenesis was thought to be a simple allergic reaction, it is now clear that it is a multifocal disease consisting of abnormalities of the horny cell layer, inflammation mainly caused by a type 2 immune reaction, and pruritus [11]. Among these, it has been identified that the interleukin (IL) -4, 13, and 31 inflammatory pathways work as a hallmark features in the pathogenesis of this disease, contributing uniquely and synergistically to immune and barrier abnormalities, as well as pruritus [12]. This finding and the advent of biotechnological breakthroughs have led to the development of treatments, e.g., monoclonal antibodies, other biologics [13], and Janus kinase (JAK) inhibitors [14]. These are a new arsenal of weapons against moderate or severe forms of atopic dermatitis.

Hand eczema is a very common disease in the general population. Although it affects limited parts of the body, it negatively impacts the quality of life and occupational performance of patients. Thyssen et al. provides a detailed summary of the management of this disease, emphasizing the importance of trigger elimination, patient education, and initial anti-inflammation treatment [15].

This Special Issue of the *International Journal of Molecular Science* entitled “Molecular Studies of Dermatitis: From Mechanism to Therapy” contains interesting papers regarding allergic contact and atopic dermatitis and hand eczema. They cover everything from mechanisms to clinical aspects, as the title suggests. I am confident that this Special Issue will help you understand that the skin is not just a barrier, but a complex immune organ.
